# SpO_2_/FiO_2_ Correlates with PaO_2_/FiO_2_ (P/F) and Radiological Biomarkers of Severity: A Retrospective Study on COVID-19 Pneumonia Patients

**DOI:** 10.3390/biomedicines13051072

**Published:** 2025-04-28

**Authors:** Alberto Marra, Vito D’Agnano, Raffaella Pagliaro, Fabio Perrotta, Ilaria Di Fiore, Antonio D’Orologio, Filippo Scialò, Angela Schiattarella, Andrea Bianco, Roberto Parrella

**Affiliations:** 1 U.O.C. Malattie Infettive ad Indirizzo Respiratorio, Cotugno Hospital, AORN dei Colli, 80131 Napoli, Italy; dr.marra@outlook.com (A.M.); roberto.parrella@ospedalideicolli.it (R.P.); 2 Department of Translational Medical Sciences, University of Campania L. Vanvitelli, 80131 Naples, Italy; fabio.perrotta@unicampania.it (F.P.); ilaria.difiore@studenti.unicampania.it (I.D.F.); dorologioantonio@gmail.com (A.D.); angela.schiattarella1@studenti.unicampania.it (A.S.); andrea.bianco@unicampania.it (A.B.); 3 U.O.C. Clinica Pneumologica L. Vanvitelli, Monaldi Hospital, AORN dei Colli, 80131 Naples, Italy; 4 Dipartimento di Medicina Molecolare e Biotecnologie Mediche, Università di Napoli Federico II, 80131 Napoli, Italy; filippo.scialo@unina.it

**Keywords:** SARS-CoV-2, COVID-19, SpO_2_/FiO_2_, SF ratio, HRCT, laboratory biomarkers

## Abstract

**Background:** In patients with COVID-19 pneumonia, the estimation of PaO_2_ represents the method of choice for monitoring a patient’s oxygenation status and assessing disease severity. The aim of this study is, therefore, to investigate the correlation between SpO_2_/FiO_2_ and PaO_2_/FiO_2_, as well as radiological and laboratory biomarkers of severity. **Methods:** In this monocentric observational, analytical, retrospective large cohort study, consecutive patients with a confirmed diagnosis of pneumonia from SARS-CoV-2, hospitalized at the Cotugno Hospital—AORN dei Colli—of Naples, between 1 September 2020 and 28 February 2022 were considered for study inclusion. Patients with missing data were excluded. **Results:** We included 585 patients (median age 63 [22–95]). Mean PaO_2_/FiO_2_ was 203 [66–433], whilst mean SpO_2_/FiO_2_ was 240 [81–471]. We found that P/F ratio could be predicted from S/F ratio, as described by the linear regression equation (P/F = 13.273 + 0.790 × S/F). In addition, we found that SpO_2_/FiO_2_ ratio significantly correlated with HRCT score and laboratory markers of severity, including IL-6, D-Dimer, and NLR. **Conclusions**: SpO_2_/FiO_2_ ratio represents a highly useful resource as a valid surrogate of P/F ratio in patients with COVID pneumonia, also correlating with other biomarkers of severity, such as HRCT score and key laboratory markers.

## 1. Introduction

Coronavirus disease 2019 (COVID-19) represents a highly infectious viral illness caused by severe acute respiratory syndrome coronavirus 2 (SARS-CoV-2). With more than 18 million estimated deaths worldwide, COVID-19 is characterized by an extremely heterogenous clinical scenario ranging from an asymptomatic course to severe respiratory failure requiring mechanical ventilation, septic shock, and multiorgan failure [1,2,3]. Despite the estimation of partial pressure of oxygen (PaO_2_) obtained through arterial blood gas analysis being accurate in assessing and monitoring patients’ oxygenation status, the widespread adoption of a more accessible peripheral oxygen saturation (SpO_2_) during the COVID-19 pandemic has encouraged its use for the early identification of more severe diseases [4,5]. Preexisting data obtained before the COVID-19 era have documented the correlation between SpO_2_ by pulse oximetry/FiO_2_ (SF) ratio and P/F ratio. Likewise, a large observational cohort study reported that patients with ARDS diagnosed by S/F ratio have comparable symptoms and outcomes to patients diagnosed with the P/F ratio [6,7]. With regard to COVID-19 patients, Bonaventura et al. have found that S/F ratio can be considered a reliable surrogate of P/F in patients with ARDS, helping clinicians in both diagnosis and management, particularly in non-ICU settings [8]. We thereby conducted a retrospective study to evaluate the correlation of S/F with P/F in our cohort of patients admitted in both ICU and non-ICU departments. We secondly aimed to investigate whether S/F ratio correlated with radiological and laboratory markers of disease activity employed during COVID-19.

## 2. Materials and Methods

### 2.1. Study Design, Patient Court, and Data Collection

This is a monocentric observational, analytical, retrospective large cohort study with longitudinal follow-up on 4614 patients (20% of regional hospitalizations), with a confirmed diagnosis of interstitial pneumonia from SARS-CoV-2, hospitalized at the P.O. Cotugno of the Hospital of the Hills of Naples, Regional Reference Hospital for Infectious Diseases, in the interval between 1 September 2020 and 28 February 2022, divided into 4 different periods. Two waves of high incidence (High Waves) were interspersed with 2 phases of low incidence (Low Waves). In particular, the demographic, clinical radiological data, biomarkers of inflammation, and vaccination status in addition to hospitalization times, delay in swab negativization, and hospitalization delay with respect to the onset of symptoms or the presumed date of the infection were analysed at the time of hospitalization. The medical records of patients hospitalized in the first pandemic period have been excluded from the analysis in order to standardize the methods of therapeutic access of patients to the emergency room, both by local medicine and general practitioners, in the period between the contagion and hospitalization of the same patients. The cohort included 1956 patients diagnosed with COVID-19 according to the criteria established by the World Health Organization (WHO) by a real-time reverse transcription polymerase chain reaction (RT-qPCR) test taken from a nasopharyngeal sample. The data were collected at the time of admission to the hospital, with the information recorded in electronic health records (EHR). Demographic data, comorbidities, laboratory tests, and radiographic images were extracted from electronic medical records. Laboratory tests included complete blood count, coagulation profile, serum biochemical tests, myocardial enzymes, inflammatory markers, cytokines, and lymphocyte subgroups. Data on the use of antivirals and immunomodulatory agents have also been recorded. The severity of the disease upon hospitalization has been classified according to WHO guidelines. As an alternative specification for the comorbidity score, we used the Charlson Index [9]. The study was conducted in accordance with the Declaration of Helsinki. As this was a retrospective analysis, ethical review and approval were waived for this study due to the non-interventional retrospective study design. The conduct of the research and the dynamics of the study were carried out in accordance with the Strengthening of the Observational Studies Report in Epidemiology Guidelines (STROBE) [10].

### 2.2. Variables

Arterial blood gas measurements and SpO_2_ data were collected at similar time points. Arterial blood sampling was performed through either a radial or brachial artery puncture. Arterial blood gas analysis was carried out with GEM^®^ Premier™ 5000 (Werfen; Bedford, MA, USA), whilst pulse oximetry was performed through PalmSAT 2500 Series (Nonin; Plymouth, MN, USA) using either fingertips or the ear lobe according to each patient’s features. Patients received oxygen therapy with different interfaces according to their baseline SpO_2_ and their different clinical conditions to achieve the target SpO_2_ (92–94%, 92% in COPD patients). The SF ratio value was obtained by correcting the saturation for the alveolar oxygen pressure based on the support used by the patient at the time of measurement (room air, nasal 2–15 L, mask 5–10 L, reservoir 8–15 L, venture 8–15 L), and at the same time performing the blood gas analysis. None of the patients at the time of the survey was undergoing high-flow oxygen therapy. The cohort was divided into 3 groups according to different methods in which the respiratory functional parameters were evaluated at the time of hospitalization: PaO_2_/FiO_2_ (P/F), SaO_2_/FiO_2_ (S/F), or PaO_2_/FiO_2_ and SaO_2_/FiO_2_ (P/F, S/F). Chest CT is was performed in all patients and lung damage assessment was evaluated with a total severity score ranging from 0 to 20, according to Chung and colleagues; each of the five lung lobes was scored for the degree of involvement: score 0 (no involvement; 0%), score 1 (minimal involvement; 1–25%), score 2 (mild involvement; 26–50%), score 3 (moderate involvement; 51–75%), and score 4 (severe involvement; 76–100%) [11,12]. All laboratory markers were evaluated using the first result obtained at admission.

### 2.3. Statistical Analysis

Data obtained from the study were included in a Microsoft Excel database. All statistical analyses were performed using SPSS v.27 software. The Shapiro–Wilk test was used to assess the normality of data distribution; based on the results, either the mean (for normally distributed variables) or the median (for non-normally distributed variables) was reported. Dependent and independent continuous variables were expressed as means with standard deviation or median with range and analysed with Student’s *t*-test or the Mann–Whitney test. The categorical variables were presented as a number or proportions and compared with the chi-square test or with Fisher’s exact test. The correlation between P/F and S/F ratios was analysed using Spearman’s correlation analysis. The linear relations were analysed using Pearson’s correlation coefficient (r), linear regression and, goodness-of-fit (adjusted R^2^). Linear regression modelling was utilized to compare the relationship between P/F and S/F ratios. The ROC curves were used to evaluate the prognostic value of the various parameters. For the verification of the validity of the hypothesis, we used the analysis of the variance (ANOVA) and the Fisher’s exact test. A priori sample size estimation was performed to detect a moderate correlation (r = 0.3) with 80% power and a two-sided significance level (α) of 0.05 with a minimum of 84 patients required. Differences with *p* < 0.05 were considered statistically significant. No imputations of the data were made.

## 3. Results

Out of 4614 patients who were analysed, 585 patients admitted to the UOC Malattie Infettive ad Indirizzo Respiratorio, Cotugno Hospital, AORN Ospedali dei Colli, Napoli, Italy, had both P/F ratio and S/F ratio available for respiratory function evaluation and were consecutively enrolled. Patients who had only P/F ratio or S/F ratio were excluded. Patients’ characteristics are reported in Table 1. Briefly, the average number of days that patients spent in the hospital was 19 (±11). SARS-CoV-2 nasal swab remained positive for a mean of 28 days (±10). Concurrent cardiac diseases were present in 345 patients (59%), whilst 59 (10%) patients had pre-existing pulmonary diseases. Other relevant comorbidities included metabolic (22.6%), chronic renal (9.4%), and neoplastic (9.1%) diseases. One hundred eighty-six (31.8%) patients succumbed to COVID-19.

**Figure 1:**
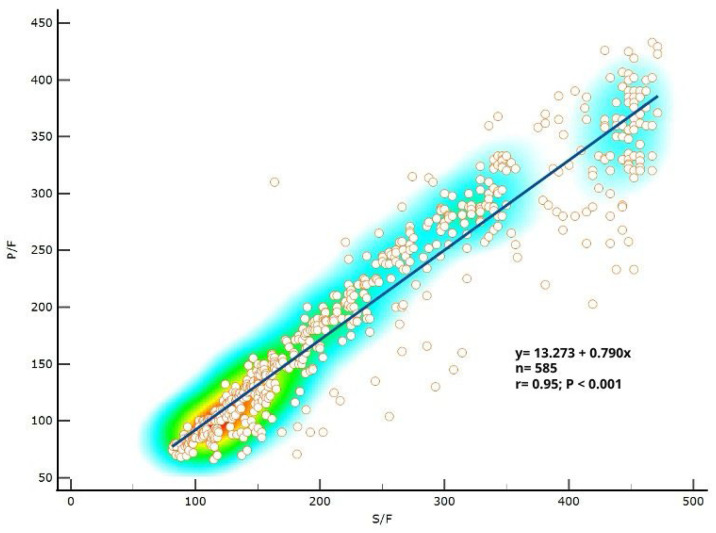
Correlation between S/F ratio and P/F ratio.

Concerning oxygen status, patients were graded into four classes—from 0 to 4—according to P/F value at admission. The mean P/F ratio was 203 (±98) (Figure 1). One hundred fourteen patients (19.5%) had a P/F ratio higher than 300, 146 pts (25%) had a P/F ratio between 300 mmHg and 200 mmHg (P/F class 1), 217 pts (37.1%) had a P/F ratio between 200 mmHg and 100 mmHg (P/F class 2), and 108 pts (18.5%) had a P/F ratio less than 100 mmHg (P/F class 3). Four hundred eighty-six (83.2%) patients received oxygen support. The median S/F ratio was 204 [81–471] at admission.

### 3.1. P/F Ratio Can Be Predicted Well from S/F Ratio in Patients with COVID-19

**Figure 2:**
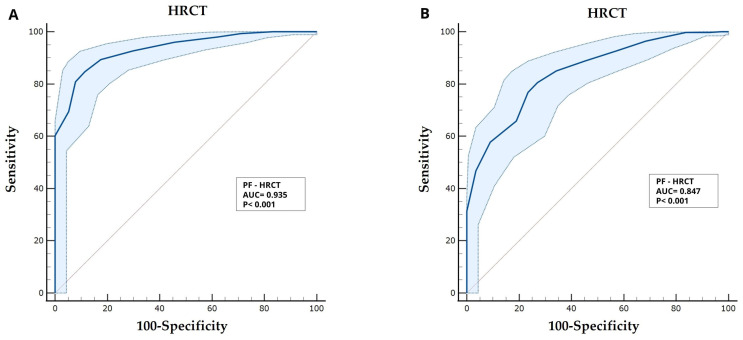
Receiver operator characteristic (ROC) curve analyses: (**A**) the predictive role of P/F ratio towards the HRCT score; (**B**) the predictive role of S/F ratio towards the HRCT score.

We compared the S/F ratio and the P/F ratio in the cohort of enrolled patients. We found that P/F ratio could be predicted from S/F ratio, as described by the linear regression equation (P/F = 13.273 + 0.790 × S/F) (Figure 2). According to this equation, an S/F ratio of 300, 200, 100 corresponds to a P/F ratio of 250, 171, 92, respectively (*p* < 0.001).

### 3.2. S/F Ratio Significantly Correlated with COVID-19 Severity Based on P/F Ratio

To correlate S/F ratio value at admission with the classes of severity based on P/F ratio, we obtained four classes of severity according to S/F ratio. Based on the results stemming from the linear regression equation, and as demonstrated by the ROC curve, a significant correlation has been found between S/F ratio and the severity of COVID-19 based on P/F ratio. In detail, patients belonging to class 0, 1, 2, or 3 had an S/F ratio of more than 350 (S/F class 0), between 236 and 350, between 123 and 236, and less than 123, respectively.

### 3.3. Severity of Diseases Based on Both P/F and S/F Did Significantly Correlate with HRCT Score

The mean HRCT score was 13 (DS: ±4). Three different classes of severity have been identified according to HRCT score: mild (HRCT score: 2–8), moderate (HRCT score: 9–14), and severe (HRCT score: 15–20). Patients who were admitted with an HRCT score between 2 and 8 had a mean P/F of 319.48 (Figure 2A), whilst for patients with a score between 15 and 20, it was 111.70 (Figure 2B). AUC curves showed that both P/F ratio (Figure 2A) [AUC = 0.834; Sensitivity = 71.92%; Specificity = 82.46%; *p* < 0.001; Youden index = 0.5437; *p* < 0.001] and S/F ratio [AUC = 0.847; Sensitivity = 80.59%; Specificity = 72.97%; Youden index = 0.5356; *p* < 0.001] (Figure 2B), significantly correlated with HRCT severity score.

### 3.4. Laboratory Findings on Admission

**Figure 3:**
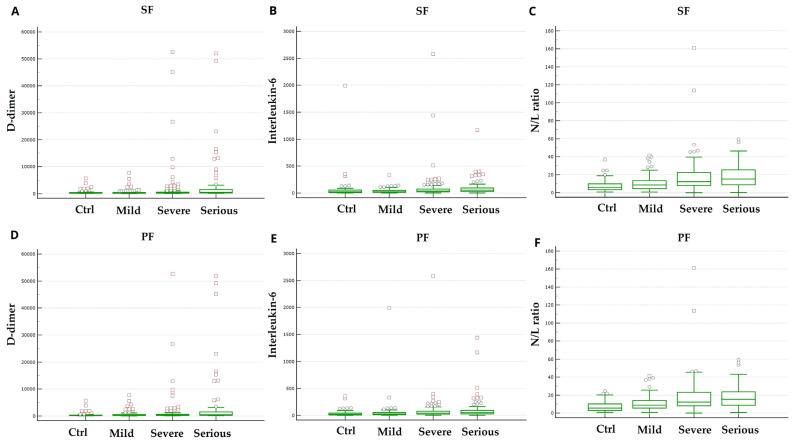
Box plots of mean serum concentrations of D-Dimer (**A**,**D**), IL-6 (**B**,**E**), and NLR value (**C**,**F**) based on SF (**A**–**C**) and PF (**D**–**F**) ratio, respectively. *p* < 0.001.

Laboratory biomarkers have been analysed at admission and displayed in Table 2. Overall mean haemoglobin was 140 g/L (±2.1). Serum concentrations of both IL-6 and D-Dimer were found to be significantly higher in patients with more severe disease based on both SF and PF ratios. Likewise, NLR value was significantly higher in patients with more severe disease compared to patients with less SF as well as PF ratio at admission (Figure 3).

## 4. Discussion

The SARS-CoV-2 pandemic represented a real challenge for healthcare systems worldwide. According to National Institutes of Health (NIH) guidelines, five classes of severity have been identified, ranging from asymptomatic to critical illness with acute respiratory failure, septic shock, and/or multiple organ dysfunction [13]. In critical patients, acute respiratory distress syndrome (ARDS) may eventually develop with dramatic consequences in terms of mortality [14]. In this scenario, clinicians have been forced to use quick and minimally invasive modalities for screening more severe COVID-19 and monitoring patients. Despite blood gas analysis representing the method of choice for oxygen status assessment, evidence has supported pulse oximetry as a reliable and rapid tool for accurately addressing this clinical situation [15,16,17]. In this respect, our results showed a strong positive correlation between S/F and P/F ratio amongst patients with COVID-19 in both ICU and non-ICU settings. Specifically, in line with our results, P/F ratio could be significantly predicted by S/F ratio at admission, according to the linear regression equation (P/F = 13.273 + 0.790 × S/F).

The advantages of using SpO_2_ over blood gas analysis are recognized in several clinical settings: pulse oximetry is rapid, non-invasive, non-painful, and can provide continuous data [18,19,20]. However, SpO_2_ may differ from SaO_2_. In addition, it has been reported that SpO_2_ accuracy may further decrease when SaO_2_ levels drop to less than 80% [21]. Interestingly, our results showed that S/F ratio also strongly correlated with P/F ratio in patients with severe COVID-19 whose P/F ratio is less than 100 mmHg. These data are in line with the aforementioned study by Bonaventura and colleagues, which showed significant accuracy of SpO_2_ in also detecting severe ARDS—defined as P/F ratio ≤ 100 mmHg—among COVID-19 patients in a non-ICU setting, with an S/F cut-off ≤ 178% (specificity: 98.4%; sensitivity: 90.8%) [8]. We confirmed the results obtained by the authors and at the same time also demonstrated the accuracy of S/F ratio in an ICU setting.

The importance of chest imaging during COVID-19 pandemia is undeniable. It was crucial, not only for diagnosis of SARS-CoV-2-related pneumonia and lung involvement assessment but also guided therapeutic approaches and was used for assessing response to treatment [22]. However, chest high-resolution computed tomography (HRCT) is undoubtedly not indicated for all patients infected by SARS-CoV-2. Risk stratification is of paramount importance for avoiding unnecessary tests and costs [23,24,25]. We have demonstrated that pulse oximetry-assessed S/F ratio significantly correlated with HRCT severity score, supporting its role as a rapid and accurate tool in COVID-19 assessment, from mild to severe disease. Our results are in line with previously published studies which have demonstrated an inverse correlation between oxygen saturation and CT severity score in a cohort of 305 SARS-CoV-2 patients [26,27]. However, to our knowledge, our study is the first to demonstrate that S/F ratio—as well as P/F ratio—is related to HRCT severity.

As for other viral infections, the role of the immune system is crucial for controlling infection spread. However, an exuberant inflammatory response has emerged to be related to poor outcomes in patients infected with SARS-CoV-2 [28,29,30]. The predictive value of laboratory markers, such as D-dimer NLR, and KL-6, in patients with COVID-19 infection has been widely investigated and demonstrated [12,31,32,33,34,35]. Compatible with these results, our data confirmed a significant correlation between some laboratory biomarkers and the severity of disease. Moreover, we have demonstrated that inflammatory markers, in particular IL-6 and D-dimer, were remarkably correlated with disease severity based on SF ratio.

We recognize that the present study has some limitations. Several biases may reduce pulse oximeter accuracy. These include pigmentation of the skin, motion artifact, poor peripheral perfusion, hypotension, changes in systemic vascular resistances, scleroderma, and nail polish. Causes of SpO_2_ underestimation, such as skin pigmentation, have not been investigated amongst patients suitable for the study and consequently are not considered as exclusion criteria. In addition, the monocentric and retrospective design of the study represent two important limitations.

## 5. Conclusions

The S/F ratio (SpO_2_/FiO_2_) emerges as a reliable, non-invasive surrogate for the P/F ratio (PaO_2_/FiO_2_) in the assessment of respiratory function in patients with COVID-19 pneumonia. Given its accessibility and ease of measurement, the S/F ratio offers a practical tool for clinical decision-making, particularly in resource-limited settings or in circumstances where arterial blood gas analysis is not readily available.

In our study, the S/F ratio demonstrated a significant correlation, not only with the P/F ratio, but also with other established indicators of disease severity, including the high-resolution computed tomography (HRCT) severity score and key laboratory biomarkers. These findings support the role of the S/F ratio as an integrated marker of both pulmonary and systemic disease burden in COVID-19. Future prospective studies are needed to validate the diagnostic and prognostic utility of the S/F ratio in broader populations, including patients with pneumonia of non-COVID aetiology. In addition, further research should explore the potential of S/F ratio-based thresholds in guiding treatment strategies and monitoring disease progression in different clinical settings.

## Figures and Tables

**Table 1:** Baseline characteristics of the patients.

Variables		
Total patients		n = 585
Gender (male/female)		388/197 (66.3%/33.7%)
Age (Years)		65 [22–95]
Hospitalization (days)		19 [0–78]
Onset-to-door (days)		7 [1–37]
Swap Positivity (Days)		26 [6–80]
Body Temperature (°C)		36.9 [35.5–39]
Exitus (healed/deceased)		399/186 (68.2%/31.8%)
PaO_2_/FiO_2_ (P/F)		203 [66–433]
P/F Severity	>300	19.5%
	200–300	25%
	100–200	37.1%
	<100	18.4%
Sat.O_2_ (%)		91 [50–100]
Sat.O_2_/FiO_2_ (S/F)		240 [81–471]
HRCT (score)		13 [2–20]
HRCT Severity	2–8	19.5%
	9–14	41.9%
	15–20	38.6%
Comorbidity (0, 1, 2, 3 or more)	0	24.8%
	1	41.8%
	2	23.3%
	≥3	10.1%
Cardiac disease		59%
Metabolic disease		22.6%
Pulmonary disease		10%
Chronic Renal Insufficiency		9.4%
Cancer		9.1%

**Table 2:** Laboratory biomarkers at admission according to S/F ratio.

	SpO_2_/FiO_2_ >350 Class 0	SpO_2_/FiO_2_ 236–350 Class 1	SpO_2_/FiO_2_ 123–236 Class 2	SpO_2_/FiO_2_ <123 Class 3
D-dimer (ng/mL)	240.0 [72–5652]	232,000 [16.0–7741.0]	320.0 [63.0–52,673.0]	514.5 [144.0–52,005.0]
Ferritin (ng/mL)	613.0 [88–3217]	786.5 [58.0–10,601.0]	1020.0 145.0–8360.0]	875.0 [139.0–60,219.0]
Haemoglobin (g/L)	140 [8–175]	140 [84–187]	142 [74–180]	136 [56–184]
IL6/Lymph (pg/mL·cells)	30.1 [0.98–6988]	31.0 [1.5–906.3]	58.1 [2.9–2875.3]	79.6 [1.3–2309.5]
IL2R (IU)	859.0 [127–17,328]	965,500 [296.0–7050.0]	1061.0 [100.0–6781.0]	1296.0 [95.0–7338.0]
IL-6 (pg/mL)	24.2 [2–1995]	24.7 [2.0–332.0]	38.3 [2.0–2580.0]	54.1 [2.0–1167.0]
NLR	5.9 [0.7–36.9]	8.6 [0.7–41.5]	12.3 [0–161.1]	15.1 [0.1–59.4]
RCP (mg/dL)	5.4 [0.4–29]	5.8 [0.4–213.0]	8.5 [0.4–124.0]	9.0 [0.4–26.6]
Platelets (×10^3^/uL)	193.0 [44–634]	211.0 [13.3–741.0]	225.0 [51.0–565.0]	241.0 [5.3–647.0]
PCT (ng/mL)	0.07 [0.02–8.2]	0.08 [0.006–55.5]	0.13 [0.006–19.7]	0.230 [0.03–40.5]
Temperature (°C)	36.5 [35.8–38.5]	36,800 [35.5–39.5]	36.8 [36.0–38.5]	37.6 [0.03–40.5]
Comorbidities				
Cardiovascular	62 (54%)	74 (50%)	130 (59%)	65 (60%)
Diabetes	24 (21%)	28 (19%)	44 (20%)	31 (28%)
Neoplastic	10 (9%)	9 (6%)	21 (10%)	11 (10%)
Respiratory	12 (10%)	10 (7%)	21 (10%)	13 (12%)
Renal	7 (6%)	14 (10%)	15 (7%)	17 (16%)

## Data Availability

Data can be shared upon request.
